# Identification of Active Site Residues of the Siderophore Synthesis Enzyme PvdF and Evidence for Interaction of PvdF with a Substrate-Providing Enzyme

**DOI:** 10.3390/ijms22042211

**Published:** 2021-02-23

**Authors:** Priya Philem, Torsten Kleffmann, Sinan Gai, Bill C. Hawkins, Sigurd M. Wilbanks, Iain L. Lamont

**Affiliations:** 1 Department of Biochemistry, School of Biomedical Sciences, University of Otago, Dunedin 9054, New Zealand; naimamode@gmail.com (P.P.); torsten.kleffmann@otago.ac.nz (T.K.); sigurd.wilbanks@otago.ac.nz (S.M.W.); 2 Centre for Protein Research, University of Otago, Dunedin 9054, New Zealand; 3 Department of Chemistry, Division of Sciences, University of Otago, Dunedin 9054, New Zealand; gaisinan@126.com (S.G.); bhawkins@chemistry.otago.ac.nz (B.C.H.)

**Keywords:** pyoverdine, siderophore, formyltetrahydrofolate, hydroxamate, siderosome, multienzyme complex

## Abstract

The problematic opportunistic pathogen *Pseudomonas aeruginosa* secretes a siderophore, pyoverdine. Pyoverdine scavenges iron needed by the bacteria for growth and for pathogenicity in a range of different infection models. PvdF, a hydroxyornithine transformylase enzyme, is essential for pyoverdine synthesis, catalysing synthesis of formylhydroxyornithine (fOHOrn) that forms part of the pyoverdine molecule and provides iron-chelating hydroxamate ligands. Using a mass spectrometry assay, we confirm that purified PvdF catalyses synthesis of fOHOrn from hydroxyornithine and formyltetrahydrofolate substrates. Site directed mutagenesis was carried out to investigate amino acid residues predicted to be required for enzymatic activity. Enzyme variants were assayed for activity in vitro and also in vivo, through measuring their ability to restore pyoverdine production to a *pvdF* mutant strain. Variants at two putative catalytic residues N168 and H170 greatly reduced enzymatic activity in vivo though did not abolish activity in vitro. Change of a third residue D229 abolished activity both in vivo and in vitro. A change predicted to block entry of N^10^-formyltetrahydrofolate (fTHF) to the active site also abolished activity both in vitro and in vivo. A co-purification assay showed that PvdF binds to an enzyme PvdA that catalyses synthesis of hydroxyornithine, with this interaction likely to increase the efficiency of fOHOrn synthesis. Our findings advance understanding of how *P. aeruginosa* synthesises pyoverdine, a key factor in host–pathogen interactions.

## 1. Introduction

**Figure 1:**
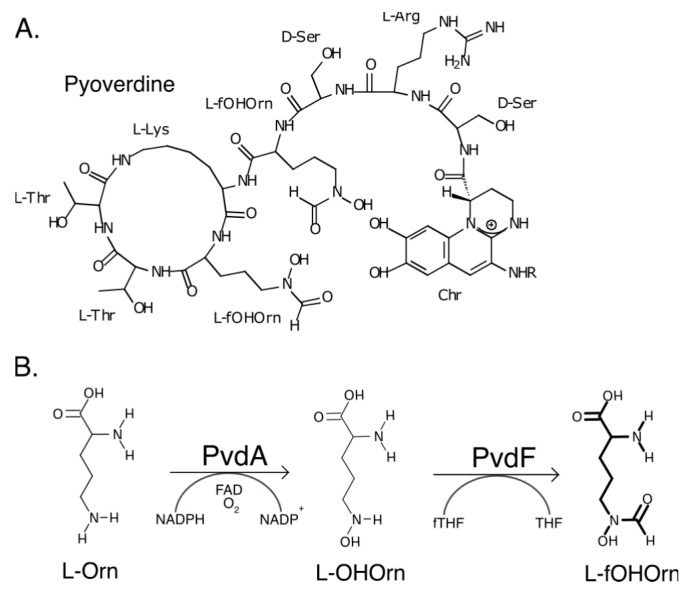
Pyoverdine structure and synthesis of formylhydroxyornithine. (**A**) Pyoverdine from *P. aeruginosa* PAO1. (**B**) Synthesis of formylhydroxyornithine (fOHOrn) from l-ornithine (l-Orn). PvdA catalyses hydroxylation of l-Orn to l-OHOrn which is then formylated by PvdF with N^10^-formyltetrahydrofolate (fTHF) as the co-substrate to give l-fOHOrn and tetrahydrofolate (THF).

Siderophores are low molecular weight (200–2000 Da) ferric ion-specific chelating agents secreted by bacteria and fungi in order to obtain iron required for growth [1]. Pyoverdines are yellow green fluorescent siderophores secreted by Pseudomonads, including the opportunistic pathogen *P. aeruginosa*. In addition to being iron transporters, pyoverdines are inducers of virulence factor production [2]. Absence of pyoverdine attenuates virulence in multiple infection models [3,4,5,6]. Pyoverdines consist of a 2,3-diamino-6,7-dihydroxyquinoline fluorophore that gives yellow green fluorescence, a variable acyl side chain attached to the 3-amino group of the fluorophore, and a strain-specific peptide backbone of 6–12 amino acids (Figure 1), including D-isomers and other non-proteinaceous amino acids [7,8]. One of these is N^5^-l-formylhydroxyornithine (fOHOrn), present in the pyoverdine of *P. aeruginosa* reference strain PAO1. Synthesis of fOHOrn requires the PvdA and PvdF enzymes. PvdA catalyses conversion of l-ornithine (Orn) to L-N^5^-hydroxyornithine (OHOrn) [9]. PvdF formylates OHOrn, with N^10^-formyltetrahydrofolate (fTHF) as the co-substrate, to produce fOHOrn (Figure 1) [10,11]. fOHOrn is then incorporated into the pyoverdine precursor peptide by the non-ribosomal peptide synthetases (NRPSs) PvdI and PvdJ [12,13,14]. A *P. aeruginosa pvdF* mutant failed to make pyoverdine, demonstrating that synthesis of fOHOrn is essential for pyoverdine production [10]. Formyltransferases are also required for generation of fOHOrn for siderophore synthesis in other bacteria [15,16].

The structure of PvdF was recently reported [11]. The enzyme is a monomer adopting a formyltransferase fold also present in other fTHF dependent enzymes, with a central 7-stranded sheet surrounded by helices and loops. Although a THF analog was observed in this crystal structure, the authors suggest that it is bound outside the active site. Therefore, no direct evidence exists for which residues participate in substrate binding and catalysis. The closest characterised structural and functional homologue of PvdF is glycinamide ribonucleotide transformylase (GART) from *E. coli* [10,11]. GART catalyses the formylation of N^1^-(5-phospho-d-ribosyl)glycinamide (GAR) with fTHF as the formyl donor as part of the de novo purine synthesis pathway. The crystal structure of *E. coli* GART in complex with GAR and a fTHF analogue (Figure S1) [17] identified substrate-binding and catalytic amino acid residues and allowed proposal of a catalytic mechanism. The effects of changes at key residues were consistent with the proposed mechanism and enhanced understanding of substrate binding and catalysis [18,19]. We used sequence alignment and the superposition of PvdF and GART structures to identify conserved residues that are likely to be active site residues of PvdF.

There is evidence that enzymes involved in pyoverdine synthesis, including PvdA and NRPS enzymes, form a multienzyme complex that has been termed a siderosome [20,21]. Multienzyme complexes enhance the efficiency of biosynthetic pathways by allowing the substrates to channel from one enzyme to the next without release into the cellular matrix, increasing the local concentration of substrate and protecting any unstable compounds from cellular conditions that may result in side reactions [22]. OHOrn, the product of PvdA and substrate of PvdF, may be prone to side reactions [23,24]. Prompt formylation following OHOrn formation would be facilitated if PvdF was part of the pyoverdine siderosome. However, whether PvdF is part of such a complex, or physically interacts with other pyoverdine synthesis enzymes, has not been investigated to date.

The aims of this research were to investigate the effects of changes to predicted active site residues of PvdF on enzyme activity both in vitro and in vivo and by co-purification to detect interactions between PvdF and PvdA.

## 2. Results

### 2.1. Purification and Activity of PvdF

Prior to this study, PvdF had been hypothesized to catalyse the conversion of OHOrn to fOHOrn with fTHF as the co-substrate. To test this hypothesis, recombinant PvdF was overexpressed fused to an *N*-terminal (His_6_PvdF) or *C*-terminal (PvdFHis_6_) hexahistidine tag. Two different fusions were engineered in case a fusion at either the *N*- or *C*-terminus interfered with enzymatic activity. The recombinant proteins were purified using Ni^2+^ affinity resin and size exclusion chromatography (Figure S2). Soluble and homogenous proteins were obtained, with PvdF purifying as a monomer as also reported by others [11].

**Figure 2:**
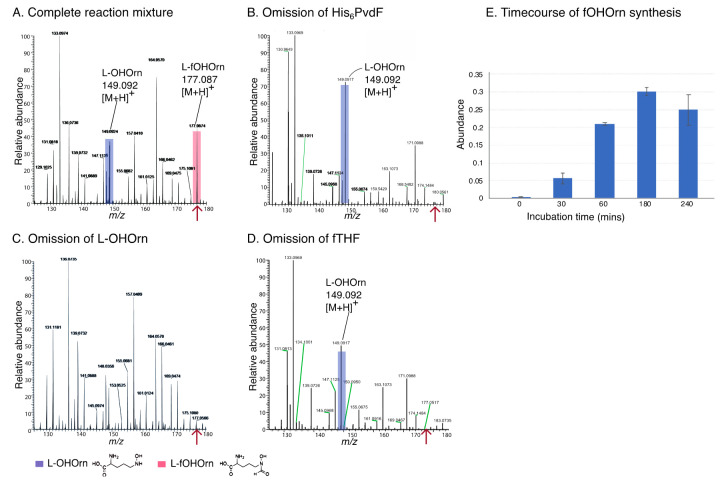
PvdF catalyses synthesis of fOHOrn from OHOrn and N^10^-formyltetrahydrofolate (fTHF). Reaction mixtures containing OHOrn (hydroxyornithine), N^10^-fH^4^F and His_6_PvdF were incubated at 30 °C for 3 h and analysed by direct injection mass spectrometry. Spectra present relative peak intensities of ionised molecules in a range of mass to charge ratios (*m*/*z*) from 125 to 185. (**A**) His_6_PvdF reaction showing peaks corresponding to OHOrn ([M+H]^+^ 149.0921 ± ppm; blue) and fOHOrn (formylhydroxyornithine) ([M+H]^+^ 177.0870 ± 3 ppm; red). (**B**) Negative control with omission of His_6_PvdF. (**C**) Negative control with omission of OHOrn. Note the background peak at m/z 177.0566 is not related to the fOHOrn peak at *m*/*z* 177.0870 (delta *m*/*z* > 170 ppm). (**D**) Negative control with omission of fTHF. (**E**) Timecourse of fOHOrn synthesis. Assays were set up in technical triplicates at 0, 30, 60, 180 and 240 min. Abundances of L-fOHOrn were obtained and the average of three technical replicates is shown as a function of incubation time. The error bars are standard errors of the mean. Spectra are representative of at least three independent enzyme preparations.

Purified PvdF was tested for activity with substrates OHOrn and fTHF. The presence of OHOrn ([M+H]^+^ = 149.0921) and the predicted reaction product fOHOrn ([M+H]^+^ = 177.0870) was detected using direct injection mass spectrometry (Figure 2A and Figure S3A). The product was not made in the absence of PvdF or either of the substrates (Figure 2B–D; Figure S3B–D). These findings indicate that both forms of recombinant PvdF are enzymatically active and were able to convert OHOrn to fOHOrn with fTHF as the co-substrate. Reactions were carried out for up to 4 h and the highest amount of fOHOrn was present after 3 h of incubation (Figure 2E). A similar assay for PvdF was developed independently and reported during the course of this research [11]. Those researchers also found that purified PvdF catalyses the formation of fOHOrn from OHOrn and fTHF substrates.

### 2.2. Active Site Prediction and Site Directed Mutagenesis

**Figure 3:**
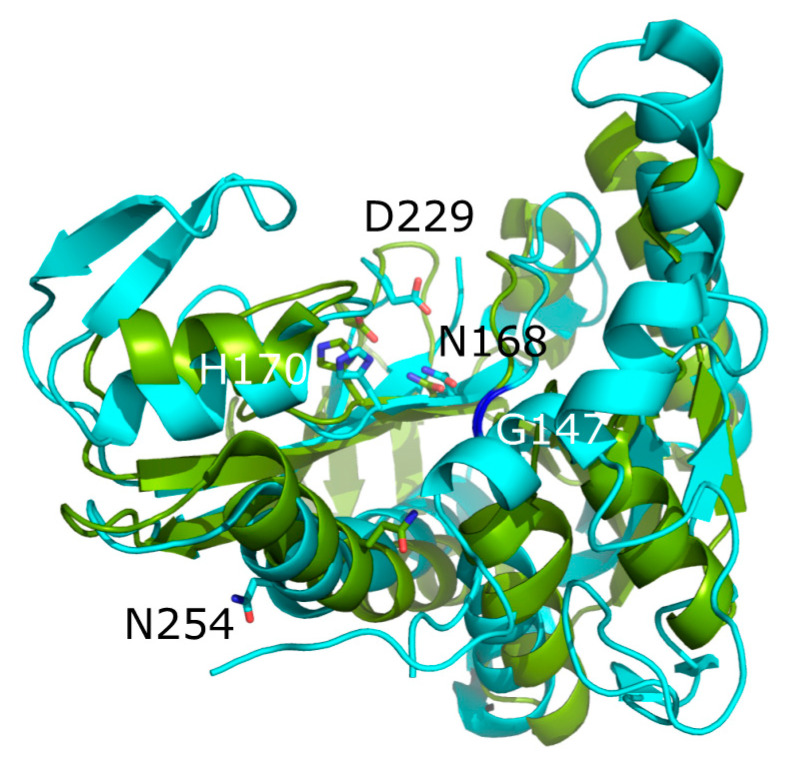
Superposition of PvdF on glycinamideribonucleotide transferase (GART). The view looks into the major cleft between domains of PvdF (cyan, chain H of PDB 6CUL). This aligns with the active site of GART (chain A of PDB 1C2T, shown in olive). Stick representations of sidechains identify the residues that were modified in PvdF and the corresponding active site residues in GART with oxygen in red and nitrogen in blue. G147 of PvdF and G 87 of GART are emphasised in dark blue. Residues are labelled with the PvdF numbering.

The likely active site of PvdF was identified by superimposing the PvdF structure on that of its closest characterised homologue, glycinamideribonucleotide transferase (GART) of *E. coli* (Figure 3) [11]. GART catalyses the conversion of N^1^-(5-phospho-d-ribosyl) glycinamide (GAR) to N^2^-formyl-N^1^-(5-phospho-d-ribosyl) glycinamide (fGAR) with fTHF as the co-substrate [25]. Engineering variants of residues in the proposed substrate binding pocket of GART identified N106, H108 and D144 residues as likely being involved in catalysis [18] (Figure S1). The corresponding residues N168, H170 and D229 are present in the proposed PvdF active site (Table 1). Furthermore, G87 in the substrate-binding pocket of GART and Q170 that forms hydrogen bonds with the substrate have corresponding residues (G147 and N254) in PvdF. These conserved residues were selected for analysis in order to explore their roles in PvdF. To facilitate comparison with GART, equivalent substitutions to those made in GART [18] (Table 1) were made. The residues and the substituting amino acids are listed in Table 1. The PvdF variants were engineered using site directed mutagenesis and were purified in the same way as wildtype PvdF (Figure S4). Circular dichroism showed that enzyme variants had similar secondary structure composition to the wildtype enzyme, indicating that the changes did not cause major changes to the protein structure (Figure S5). The α-helix and β-sheet compositions indicated by circular dichroism were consistent with that of the PvdF structure derived from X-ray crystallography [11].

### 2.3. Effects on Enzyme Variants on Protein Stability

Differential scanning fluorimetry was used to investigate the effects of mutations and substrates on the thermal stability of PvdF. Unfolding of globular proteins due to increasing temperature allows SYPRO^®^ orange dye to bind to newly exposed hydrophobic regions of the protein, leading to increased fluorescence [26]. Melting temperatures were calculated as the inflection points in melting curves, and the presence of more than one inflection was interpreted as independent melting events of multiple domains or oligomeric states [27].

**Figure 4:**
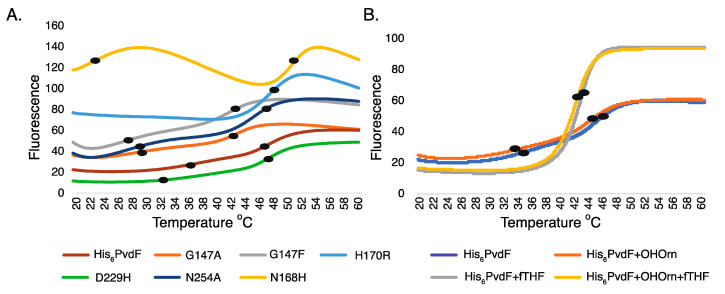
PvdF consists of two independently melting domains. (**A**) Thermal stability tests of PvdF and variants were carried out using differential scanning fluorometry. Black dots indicate inflection points that are likely to represent domain melting events. All the variants had evidence for two inflection points except H170R which showed only one. (**B**) Stability tests of His_6_PvdF in presence of fTHF (formyltetrahydrofolate) (grey and yellow curves) showed a single, cooperative melting event of large amplitude, in contrast to the two inflection points and low amplitudes observed in absence of this substrate (blue and orange curves). In contrast, the presence of OHOrn (hydroxyornithine) (grey and orange curves) did not affect melting of PvdFHis_6_. Spectra are averages of at least three independent enzyme preparations.

Two inflection points were observed with wildtype PvdF (Figure 4A), consistent with PvdF being comprised of two independent domains [11] that melt separately in this assay [28], although the lower temperature transition was broad. Two inflection points were also observed for the engineered variants G147A, G147F, N168H, D229H and N254A but only one for variant H170R (Figure 4A). Variants N168H and H170R have a high fluorescence signal at low temperature, suggesting that these changes may destabilize one domain of the enzyme.

Substrate binding can increase the stability of enzymes, resulting in increased melting temperature [29]. Addition of OHOrn did not affect the thermal shift of wild-type or variant enzymes (Figure 4B and Figure S6) indicating that this substrate was bound only weakly. In contrast, addition of fTHF resulted in a significant change in fluorescence of wild-type PvdF (Figure 4B). This indicates robust binding of this substrate to the enzyme. Variants G147A, G147F, D229H and N254A are qualitatively similar to wild-type when comparing presence and absence of fTHF (Figure S6). Addition of fTHF caused increased fluorescence of N168H and N170R at low temperatures, with a broader transition and a marked decline of fluorescence at high temperatures, consistent with reduced stability relative to wild-type enzyme.

In summary, differential scanning fluorimetry supports the existence of two independently folded domains in solution. It further shows that all variants retain a folded structure and can bind fTHF at the temperatures used for assay function, consistent with circular dichroism analysis, but variants N168H and H170R have compromised stability.

### 2.4. Functional Analysis of Enzyme Variants

**Figure 5:**
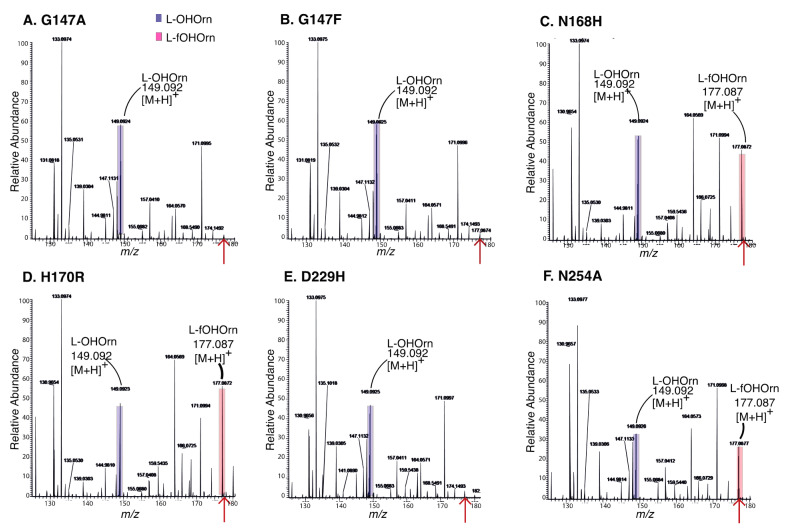
In vitro activity of PvdF enzyme variants. Reaction mixtures of PvdF variants were incubated at 30 °C for 3 h and then analysed using direct injection mass spectrometry. Spectra present relative peak intensities of ionised molecules in a range of mass to charge ratios (*m*/*z*) from 125 to 185. The detection of OHOrn (hydroxyornithine) at *m*/*z* 149.0921 ± 3 ppm and fOHOrn (formylhydroxyornithine) at *m*/*z* 177.0870 ± 3 ppm is indicated by peaks highlighted in blue and red, respectively. (**A**) G147A. (**B**) G147F. (**C**) N168H. (**D**) H170R. (**E**) D229H. (**F**) N254A. Spectra are representative of at least three independent enzyme preparations.

The PvdF variants were investigated in vivo and in vitro to determine the effects of the mutations on enzymatic activity. Enzyme reaction products were analysed qualitatively using mass spectrometry (Figure 5). fOHOrn was formed in the N168H, H170R and N254A reaction mixtures indicating that these variants retain enzymatic function whereas fOHOrn was not detected in reaction mixtures with G147A, G147F and D229H, indicating that these variants were inactive.

**Figure 6:**
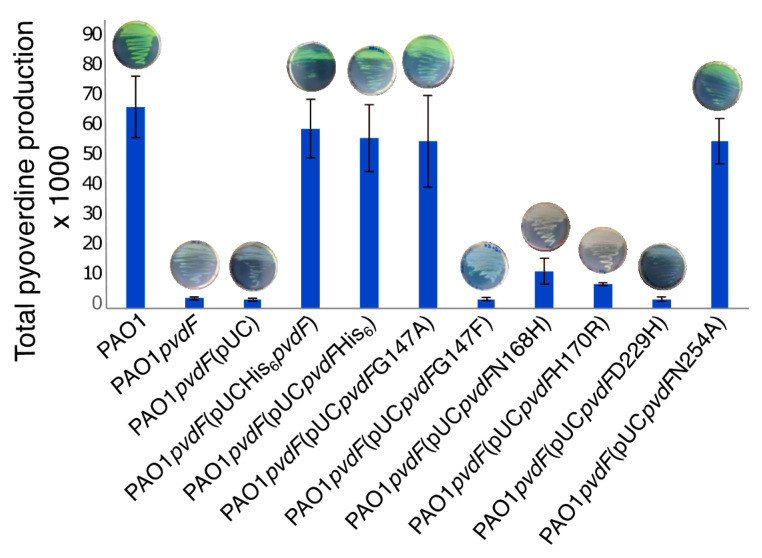
Pyoverdine production in *P. aeruginosa pvdF* containing PvdF variants. Pyoverdine production in broth culture was measured for the *P. aeruginosa pvdF* mutant containing plasmid-borne *pvdF* variants. The mean of three biological replicates along with standard error bars are shown. Agar plates, with pyoverdine giving green pigmentation, are shown above each bar.

To quantify the enzyme activities in vivo, the *pvdF* mutants encoding variant enzymes were cloned into plasmid pUCP20 with the genes being expressed from the lac promoter present in the plasmid. The resulting constructs were transformed into a *P. aeruginosa* mutant that lacks *pvdF* and so is unable to make pyoverdine. Pyoverdine production was monitored visually on agar plates and was quantified by fluorescence assay. As expected, pyoverdine production was restored to wild-type levels by the presence of unmutated *pvdF* (Figure 6). The G147A and N254A variants also restored pyoverdine to wild-type levels indicating high enzymatic activity. No detectable pyoverdine was made by bacteria containing the G147F or D229H variants. The N168H and H170R variants resulted in pyoverdine synthesis but in amounts 7–8 times lower than the wild-type.

### 2.5. PvdF and PvdA Interaction

**Figure 7:**
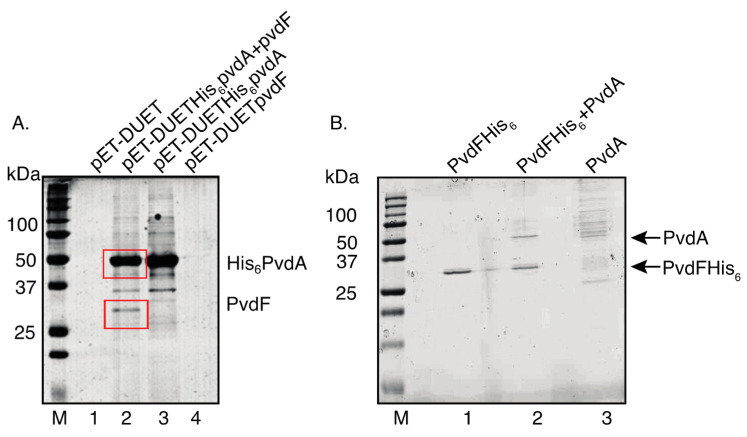
Co-purification of PvdF and PvdA (**A**) PvdF and His_6_PvdA were expressed from pET-DUET-1 in *E. coli* BL21 DE3. Following purification by nickel affinity chromatography, proteins from bacteria containing the plasmids shown were visualised by SDS-PAGE. The identities of the bands in red boxes were confirmed as His_6_PvdA and PvdF by mass spectrometry (Table S1). (**B**) PvdFHis_6_ and PvdA were expressed separately in *E. coli* BL21 DE3 and cell lysates prepared. Nickel affinity chromatography was carried out on individual lysates (PvdFHis_6_; PvdA) and following mixture of lysates (PvdFHis6 and PvdA). The eluates were analysed using SDS-PAGE. The positions of PvdFHis_6_ and PvdA proteins are indicated.

The PvdA enzyme is an ornithine monooxygenase that provides the OHOrn substrate for PvdF in vivo [9,30]. Protein–protein interactions between PvdA and PvdF would enhance channelling of OHOrn from PvdA to PvdF, maximising metabolic efficiency and minimising potential side reactions [23,24]. Co-purification (“Pull-down”) experiments were used to investigate PvdF–PvdA interaction. PvdF and His_6_PvdA were co-expressed in *E. coli* and Ni^2+^-affinity resin was used to purify His_6_PvdA. Untagged PvdF was co-purified indicating that PvdF and PvdA interact (Figure 7A).

In reciprocal experiments, purification of His_6_PvdF did not result in detectable co-purified PvdA raising the possibility that the *N*-terminal hexahistidine tag on PvdF interferes with PvdA–PvdF interactions. To test this possibility, PvdFHis_6_ that has a C-terminal hexahistidine tag and PvdA were separately expressed in *E. coli* and the soluble fractions of cell lysates were mixed. Purification of PvdFHis_6_ resulted in co-purification of PvdA (Figure 7B) confirming that PvdA and PvdF can stably interact.

## 3. Discussion

PvdF is required for pyoverdine production. As well as confirming that this enzyme catalyses the synthesis of fOHOrn from OHOrn and fTHF, our results demonstrate the importance of likely active site residues in catalysis and indicate that PvdF is likely to be part of the siderosome multienzyme complex that carries out pyoverdine synthesis.

PvdF, like other fTHF dependent enzymes, has two subdomains [11]. Thermal stability experiments revealed two protein melting events that are likely to correspond to independent melting of the two subdomains. In the presence of fTHF, one of the domains was stabilized with melting happening at a higher temperature and protein aggregation being suppressed. The presence of OHOrn did not affect protein melting curves, in either the presence or absence of fTHF. These data indicate that fTHF binds to PvdF in vitro, whereas binding of OHOrn is too transient to alter protein melting. In the crystal structure of PvdF, THF is bound away from the active site and, in this study, variants G147A and in particular G147F that are predicted to interfere with binding of THF at the active site did not prevent fTHF from affecting protein melt curves. It remains to be determined whether the effect of fTHF on protein melting is due to binding at the active site, or an alternative site on the protein.

Tetrahydrofolate dependent enzymes are well characterised in *E. coli* and human [17,18,31]. In *E. coli* GART, catalysis is proposed to involve formation of a salt bridge between ionised D144 and protonated H108 [32]. Protonated H108 is thought to act as a general acid catalyst to accelerate formation of a tetrahedral reaction intermediate. The N106 of GART stabilizes the oxyanion in the putative tetrahedral intermediate [17,31]. Substitutions D144H, H108R and N106H all resulted in inactive GART although other substitutions at these residues retained some activity in vivo [18]. In PvdF the substitutions N168H and H170R (changes of likely catalytic residues, equivalent to N106H and H108R substitutions in GART) retained some enzymatic activity, both in vitro and in vivo, contrasting with the GART findings. The in vivo assays (Figure 6) demonstrate that enzymatic activity of these two variants was much lower than that of wild-type. These findings show that likely catalytic residues N168 and H170 are important but not essential for enzymatic activity of PvdF. The in vivo findings indicate reduced catalytic activity of the N168H and H170R variants, a reduction that would not be detected by the in vitro endpoint assay. In contrast, the substitution at the likely catalytic residue D229H (equivalent to D144H in GART) had little effect on either structure or stability, but resulted in no detectable activity in vivo or in vitro showing that D229 is required for PvdF activity.

N254 of PvdF aligns with Q170 of *E. coli* GART which was proposed to be substrate binding [17]. However, PvdF containing the N254A substitution showed activity both in vivo and in vitro indicating that N254 is not required for substrate binding. G147 of PvdF is conserved with G87 of *E. coli* GART which is present in the substrate binding cleft next to the interface where substrate and co-substrate bind [31]. G147F substitution in PvdF resulted in minimal structural perturbation but no enzymatic activity, either in vitro or in vivo. The introduced phenylalanine is likely to block binding of the OHOrn substrate (Figure 3), preventing product formation. Thermal melting experiments with the G147F variant indicated that binding of fTHF was not affected by this variant. The more conservative G147A substitution was active in vivo though it did not have detectable activity in vitro. This difference likely reflects the different conditions of the in vitro assay, such as lack of interacting proteins that could provide a high local concentration of substrate at the enzyme active site.

Substrate channelling and multienzyme complexes were discovered in many cellular pathways [33,34,35,36]. The occurrence of siderophore biosynthetic enzymes as a complex could be a metabolic strategy to avoid accumulation of toxic intermediate compounds inside cells [33]. There is strong evidence for such a complex for pyoverdine synthetic enzymes, including PvdA [20,21,37]. Although it provides the fOHOrn precursor for pyoverdine synthesis, PvdF has not previously been investigated for interactions with other pyoverdine synthesis enzymes. Co-purification experiments demonstrated a stable protein–protein interaction of PvdF and PvdA (Figure 7) implying that PvdF forms part of the pyoverdine synthesis siderosome. Direct interaction with PvdA would facilitate substrate channelling so that OHOrn is promptly formylated and sequestered into the nascent pyoverdine backbone by the pyoverdine biosynthesis machinery. Failure of PvdA to co-purify with PvdF carrying an *N*-terminal hexahistidine tag suggests that the *N*-terminal region of PvdF is important for PvdF–PvdA interaction.

In conclusion, our in vitro and in vivo assays identify likely catalytic residues of PvdF and further confirms that PvdF has two domains. Our study also demonstrates that PvdF interacts with PvdA and by implication the pyoverdine siderosome. These findings provide a platform for identification of inhibitors of PvdF activity with potential to prevent pyoverdine synthesis and hence *P. aeruginosa* infections.

## 4. Materials and Methods

### 4.1. General Methods

Bacterial strains and plasmids used in this study are listed in Table S2. Bacteria were grown at 37 °C in lysogeny broth (LB) or Kings B medium [38] at 37 °C with shaking at 225 rpm or on LB agar [39]. Growth media were supplemented with ampicillin (25 µg/mL), carbenicillin (300 µg/mL) and isopropyl β-d-1-thiogalactopyranoside (IPTG) (600 µM) as required. Chemicals were purchased from Sigma-Aldrich, Merck KGAa, Darmstadt, Germany.

### 4.2. Overexpression and Purification of PvdF

The *pvdF* gene was amplified from *P. aeruginosa* genomic DNA using primers listed in Table S3. For PvdF fused to an *N*-terminal hexahistidine tag (His_6_PvdF), HindIII and BamHI restriction sites were incorporated into forward and reverse primers to enable cloning. The amplified gene was cloned into HindIII- and BamHI-digested pET-DUET1 vector that encodes an His_6_ tag located in multiple cloning site I. For PvdF fused to a *C*-terminal His_6_ tag (PvdF His_6_), *pvdF* was amplified from the *P. aeruginosa* genome using primers with NdeI and HindIII restriction sites. The amplified gene was cloned into pET21a vector that had been treated with the same restriction enzymes resulting in expression of PvdF with a *C*-terminal His_6_ tag. Plasmids were transformed into *Escherichia coli* BL21 (DE3) using the CaCl_2_ chemical transformation method [40]. PvdF was partially purified using Ni-affinity resin (IMAC) and was further purified using size exclusion chromatography (SEC). The purification was carried out at 4 °C. Overnight cultures of *E. coli* BL21(pET-DUET*his*_6_*pvdF*) or *E. coli* BL21(pET21a*pvdFhis*_6_) were diluted to OD_600_~0.15 with lysogeny broth (1L of LB) and ampicillin. The cells were grown to an OD_600_~0.6 and IPTG was added to induce protein overexpression. Incubation was continued for a further 4 h. The cells were harvested by centrifugation at 11,066× *g* at 4 °C for 25 min. The pellets were resuspended in binding buffer (20 mM sodium phosphate (pH 8.0), 300 mM NaCl) and the cells were lysed using sonication (Branson Sonifier model 350, amplitude 30–32%, 6 cycles of 8–10 s) (Emerson Electric Co., St Louis, MO, USA). Following centrifugation at 11,066× *g* at 4 °C for 25 min, the soluble fraction was used for purification by a batch method of immobilized metal affinity chromatography. The cell lysate (10 mL) was added to Ni^2+^-affinity resin (Bio-Rad, Hercules, CA, USA) (5 mL) that had been pre-equilibrated with milli Q water and wash buffer (20 mM sodium phosphate (pH 8.0), 300 mM NaCl). The mixture was incubated at 4 °C in a rotating mixture for 1 h. The supernatant was discarded following centrifugation at 2500× *g* rpm for 20 min at 4 °C in a bench top centrifuge. Two washing steps were carried out, one using binding buffer and a second using wash buffer (20 mM sodium phosphate (pH 8.0), 300 mM NaCl, 5 mM imidazole), with 6 resin volumes for each wash. Finally, the protein was recovered using elution buffer containing 20 mM sodium phosphate (pH 8.0), 300 mM NaCl, 50 mM imidazole. Partially purified His_6_PvdF was further purified by size exclusion chromatography (SEC) including exchange of the buffer to 10 mM HEPES (pH 8.0), 100 mM NaCl. The eluate was analysed using SDS-PAGE.

### 4.3. Functional Assays of PvdF

l-OHOrn was chemically synthesized using the method of [41]. N^5^-formylTHF was converted into fTHF using the method of [15]. Enzyme reactions were carried out in a volume of 50 µL using a protocol adapted from [15]. PvdF (final concentration of 25 µM) and OHOrn (final concentration of 1 mM) were added to fTHF (1.5 mM) in HEPES buffer (10 mM, pH 8.0). Reactions were incubated for up to 4 h at 30 °C and then terminated by addition of formic acid (2 µL). The samples were analysed by nanospray direct injection mass spectrometry in 5% acetonitrile and 0.1% formic acid in water at a flow rate of 0.8 µL/min using a LTQ-Orbitrap XL mass spectrometer (Thermo Fisher Scientific, Waltham, MA, USA) operated in positive ion mode. Spectra were acquired in the Orbitrap analyser in a range of mass to charge ratios (*m*/*z*) between 125 and 185 at a resolution of 100,000 at 400 *m*/*z*.

Mass spectrometry raw data were loaded into the Xcalibur 2.0.7 SP1 software (Thermo Fisher Scientific, Waltham, MA, USA) to visualise individual spectra and normalised peak intensities of the targeted analytes were extracted using the Qual Browser program 2.0.7 SP1 (Thermo Fisher Scientific, Waltham, MA, USA).

### 4.4. Active Site Prediction and Site Directed Mutagenesis

The structures of *E. coli* GART (PDB ID:1C2T) and PvdF (PDB ID: 6cul_1) were aligned using PyMOL (educational version) (https://pymol.org/2/, accessed on 22 February 2021) to predict active site amino acids of PvdF. Conserved amino acid residues corresponding to GART active site residues were selected for mutagenesis. Substituting amino acids were chosen based on previously reported mutagenesis studies on *E. coli* GART [17,18] and on size, charge and polarity of the amino acid. Possible structural changes such as steric hindrances were inspected in silico using PyMOL. A one-step site-directed mutagenesis protocol based on the Quick-change method [42] was used to create PvdF variants. pLUG-Prime vector carrying wild type *pvdF* was used as the template. Overlapping primers with intended mutations are listed in Table S3 and were designed [43] to amplify the entire vector. After PCR amplification with Phusion High-Fidelity polymerase (New England Biolabs, Ipswich, MA, USA), the solution containing both the amplicon and the wild type was treated twice with DpnI restriction enzyme to eliminate methylated template. The DpnI-treated sample was diluted to 1:5 and used for transforming *E. coli* JM83. Individual colonies were selected for plasmid recovery and mutations were confirmed by sequencing. The plasmids were then purified, the mutation-containing inserts cloned into pET-Duet using BamHI and HindIII restriction sites, and the resulting expression constructs transformed into *E. coli* BL21 (DE3) [40]. Confirmed variants were overexpressed and purified using the IMAC method in Section 4.2. Circular dichroism was used to analyse the folding nature of the variants. Samples were exchanged into a buffer containing potassium phosphate with NaF to minimize chloride ions. The samples were diluted with 25 mM potassium phosphate (pH 8.0) and 100 mM NaF to optimise signal. The spectra were then collected using a Jasco J-1500 CD Spectrophotometer (Jasco Inc, Easton, MD, USA) from 190 to 270 nm in a 1 mm pathlength cuvette at 22 °C. The data were converted into mean residual ellipticity (MRE) and the online database Dichroweb (http://dichroweb.cryst.bbk.ac.uk/html/home.shtml, (accessed on 22 February 2021)) was used to estimate secondary structure composition.

### 4.5. Thermal Stability and Functional Analysis of Variants

Differential scanning fluorimetry with the hydrophobic dye SYPRO^®^ Orange (InVitrogen/ Thermo Fisher Scientific, Waltham, MA, USA) was used to investigate thermal stability. Wild-type and variant enzymes were analysed in a LightCycler 480 (Roche, Basel, Switzerland) according to manufacturer’s protocol. Briefly, protein samples (10 µM) were prepared in HEPES buffer (10 mM, pH 8.0) (20 µL) containing Sypro Orange (2 µL of a 1:100 dilution of stock). The fluorescence was measured while increasing the temperature from 20 to 85 °C (~0.02 °C/s) using LightCycler^®^ 480. l-OHOrn (1 mM) and fTHF (1.5 mM) were added as required. Spectra of substrates alone were subtracted from the data. The data obtained were analysed using LightCycler^®^ 480 analyser for detection of melting events.

### 4.6. In Vivo Activity of PvdF Variants

DNA fragments encoding wild-type PvdF and PvdF variants that had been cloned into pET-DUET (Section 4.4) were digested and cloned into pUCP20 using XbaI and HindIII restriction sites. The pUCP20 constructs were used to transform competent *P. aeruginosa* PAO1 *pvdF* using the MgCl_2_ transformation method [44]. The transformants were spread onto LB agar supplemented with carbenicillin and were incubated at 37 °C overnight. Single colonies were picked the next day, inoculated onto King’s B agar supplemented with carbenicillin and were incubated at 37 °C overnight. Pyoverdine production was detected with the presence of yellow green fluorescent colonies.

To quantify pyoverdine production, overnight cultures were grown in King’s B broth supplemented with carbenicillin. The cultures were diluted into the same medium to OD_600_~0.001 in a 96-well plate with a final volume of 200 µL per well and with three technical replicates for each sample. The plate was then loaded into a FLUOstar Omega plate reader (BMG LABTECH, Ortenberg, Germany) and incubated at 37 °C with shaking (200× rpm). The OD_600_ and the fluorescence at excitation and emission wavelengths of 400 nm and 460 nm were recorded at every 30 min for 10 h. The fluorescence was normalized against the OD_600_ and the area under the curve was calculated in excel.

### 4.7. PvdF and PvdA Interaction

Interaction of PvdF and PvdA was investigated using co-purification “pull down” assays. PvdF and PvdA, with and without hexahistidine tags, were co-expressed or expressed separately from pET vectors in *E. coli* BL 21 (DE3) as described above (Section 4.2). All steps were carried out on ice or in a refrigerated bench top microcentrifuge. The soluble fraction was loaded onto the pre-equilibrated resin and was incubated overnight at 4 °C in a rotating mixer. The supernatant was discarded after centrifugation at 4 °C for 15 min at 8000 rpm. Wash buffer (20 mM sodium phosphate, pH 8.0, 300 mM NaCl) was added, the mixture was centrifuged for 8 min and the supernatant was discarded. Seven wash steps were incorporated using wash buffer to remove *E. coli* proteins bound non-specifically to the resin. In the third wash step imidazole (35 mM) was added to the wash buffer to eliminate non-specific binding proteins. The tagged protein and any interacting partners were eluted using 20 mM sodium phosphate, pH 8.0, 300 mM NaCl and 250 mM imidazole. The eluate was analysed using SDS-PAGE.

The interaction of PvdF and PvdA was further investigated by purifying PvdFHis_6_ and PvdA that had been overexpressed separately in *E. coli* BL 21(pET21a*pvdFhis*_6_) and *E. coli* BL 21(pET-DUET*pvdA*). The soluble fractions were mixed to 1:1 ratio and PvdFHis_6_ was purified using Ni^2+^-affinity resin following the same protocol used for His_6_PvdA and PvdF copurification. The eluate was analysed using SDS-PAGE to detect if the proteins were copurified.

## Figures and Tables

**Table 1:** Active site residues of *E. coli* GART conserved in PvdF, predicted role and substituting amino acids.

*E. coli* GART	PvdF	Predicted Role	Substituting Residue
G87	G147	l-OHOrn binding	A and F
N106	N168	Catalysis	H
H108	H170	Catalysis	R
D144	D229	Catalysis	H
Q170	N254	l-OHOrn binding	A

Abbreviations: GART, glycinamide ribonucleotide transformylase; L-OHOrn, hydroxyornithine.

## Supplementary Materials

The following are available online at https://www.mdpi.com/1422-0067/22/4/2211/s1, Table S1: Mass spectrometry analysis of protein obtained during co-purification of PvdF with His_6_PvdA, Table S2: Bacterial strains and plasmids used in this study, Table S3: Primers used in this study, Figure S1: Crystal structure of *E. coli* GART and its active site amino acids, Figure S2: Purification of His_6_PvdF using Ni^2+^-affinity resin and size exclusion chromatography, Figure S3: PvdFHis_6_ catalyses synthesis of fOHOrn from OHOrn and fTHF, Figure S4: Purification of PvdF enzyme variants, Figure S5: Circular dichroism of PvdF and variants, Figure S6: Addition of OHOrn had no effect on protein melting.
