# Supplementary material for: Identification of Active Site Residues of the Siderophore Synthesis Enzyme PvdF and Evidence for Interaction of PvdF with a Substrate-Providing Enzyme
Source: Int J Mol Sci. 2021 Feb 23;22(4):2211. doi: 10.3390/ijms22042211 (PMC7926923; doi:10.3390/ijms22042211)
Supplement: Supplementary file 1 [file ijms-22-02211-s001.pdf]

# Supplementary Tables and Figures

Identification of active site residues of the siderophore synthesis enzyme PvdF and evidence for interaction of PvdF with a substrate-providing enzyme

Priya Philem, Torsten Kleffmann, Sinan Gai, Bill C. Hawkins, Sigurd M. Wilbanks and Iain L Lamont

**Table S1. Mass spectrometry analysis of protein obtained during co-purification of PvdF with $His_6$PvdA***

| Description | Coverage | Unique peptides |
|---|---|---|
| $His_6$PvdA | 70.65 | 46 |
| PvdF | 53.45 | 32 |

*PvdF and $His_6$PvdA were co-purified following expression in *E. coli* BL21 DE3. The identities of $His_6$PvdA and PvdF proteins obtained following co-purification (Figure 7) were confirmed by mass spectrometry.

**Table S2. Bacterial strains and plasmids used in this study**

| **Bacterial strain** | **Genotype** | **Reference** |
|---|---|---|
| *Pseudomonas aeruginosa* PAO1 | wild-type | Laboratory stock |
| *Pseudomonas aeruginosa PAO1pvdF* | *pvdF* | (McMorran *et al.*, 2001) |
| *E. coli JM83* | *rpsL ara Δ(lac-proAB) Φ80dlacZΔM15* | Laboratory stock |
| *E. coli* BL21(DE3) | *fhuA2 [lon] ompT gal (λ DE3) [dcm] ΔhsdS λ DE3 = λ sBamHIo ΔEcoRI-B nt::(lacI::PlacUV5::T7 gene1) i21 Δnin5* | (Studier & Moffatt, 1986) |
| **Plasmid** | **Properties** | **Reference** |
| pLUG-PRIME | T7 promoter, *lacZα; amp*$^R$ | iNtRON Biotechnology, Inc |
| pGEM®-T Easy | T7 promoter, *lacZα; amp*$^R$ | Promega |
| pET-DUET-1 | *lacI*$^q$, T7 promoter, *colE1ori; amp*$^R$; co-expression vector | Novagen |
| pET-DUET-1*his*$_6$*pvdF* | pET-DUET-1 with *pvdF* gene in MCSI and His$_6$-tag upstream; *amp*$^R$ | This study |
| pET-DUET-1 *pvdF* | pET-DUET-1 with *pvdF* gene at MCSII; *amp*$^R$ | This study |
| pET-DUET-1 *his*$_6$*pvdF;pvdA* | pET-DUET-1 with *PvdF* gene at MCSI, His$_6$-tag upstream and *pvdA* gene in MCSII; *amp*$^R$ | This study |
| pET-DUET-1 *pvdA* | pET-DUET-1 with *pvdA* gene at MCSII; *amp*$^R$ | This study |
| pET-DUET-1 *his*$_6$*pvdA* | pET-DUET-1 with *pvdA* at MCSI and His$_6$-tag upstream' *amp*$^R$ | This study |
| pUCP20 | *lac* promoter, *lacZα; amp*$^R$; expression vector | (Schweizer, 1991) |
| pET21a | T7 promoter, pBR322 origin; *amp*$^R$; expression vector | Novagen |

**Table S3. Primers used in this study**

| Primer | Sequence (5′ – 3′) | Cloning vector |
|---|---|---|
| **DNA cloning** | | |
| pvdFforexpress | GGGGGATCCAATGACGAAAAGGAAACTGGC | MCS I pET-DUET1 |
| pvdFexpressrev | GGGAAGCTTTCAGAGCTTCTCGGCGAG | MCS I pET-DUET1 |
| pvdA For | GGGCATATGATGACTCAGGCAACTGCAACC | MCS II pET-DUET1 |
| pvdA Rev | GGGCTCGAGCGGGACATGCAACGAAAACG | MCS II pET-DUET1 |
| PriyaAfor | GGGGAGCTCCATGACTCAGGCAACTGCAACC | MCS I pET-DUET1 |
| PriyaArev | GGGGCGGCCGCGACATGCAACGAAAACG | MCS I pET-DUET1 |
| PriyaFfor | GGGCAATTGAATGACGAAAAGGAAACTGGC | MCS II pET-DUET1 |
| PriyaPvdF | GGGGACGTCTCAGAGCTTCTCGGCGAG | MCS II pET-DUET1 |
| PvdFC-FOR | GGGGTATACATGACGAAAAGGAAACTGGCC | pET-21(a) |
| PvdFC-REV | GGGAAGCTTGAGCTTCTCGGCGAGCA | pET-21(a) |
| **Site-directed mutagenesis** | | **Mutation** |
| ForPvdFG147A | TTGGACGCTCTCCTGGTCATCCTCGATGAGCTGGT | G147A |
| RevPvdFG147A | CAGGAGAGCGTCCAATACCACCACATCGGCGCCCA | G147A |
| ForPvdFG147F | TTGGACTTTCTCCTGGTCATCCTCGATGAGCTGGT | G147F |
| RevPvdFG147F | CAGGAGAAAGTCCAATACCACCACATCGGCGCCCA | G147F |
| ForFN168H | ATCATGCATATCCATCCTGGCGTGACGCGCGAGGA | N168H |
| RevFN168H | ATGGATATGCATGATCCGCCGTGCGAACGGAGCGC | N168H |
| ForFH170R | AATATCAGACCTGGCGTGACGCGCGAGGACTCGC | H170R |
| RevFH170R | GCCAGGTCTGATATTCATGATCCGCCGTGCGAAC | H170R |
| ForFD229H | GGCATCCATTCCGGCGAAGTGTTCCATGATGTG | D229H |
| RevFD229H | CGCCGGAATGGATGCCATTGTCCACATAGTGGAAC | D229H |
| ForFN254A | CGCTGGGCTAACTTCAACAACAGCCTGTTCCCG | N254A |
| RevFN254A | GAAGTTAGCCCAGCGCAGCTCGAGGATGGTGTCGT | N254A |

Primers were purchased from Integrated DNA Technologies and Macrogen. Introduced restriction sites are under lined.

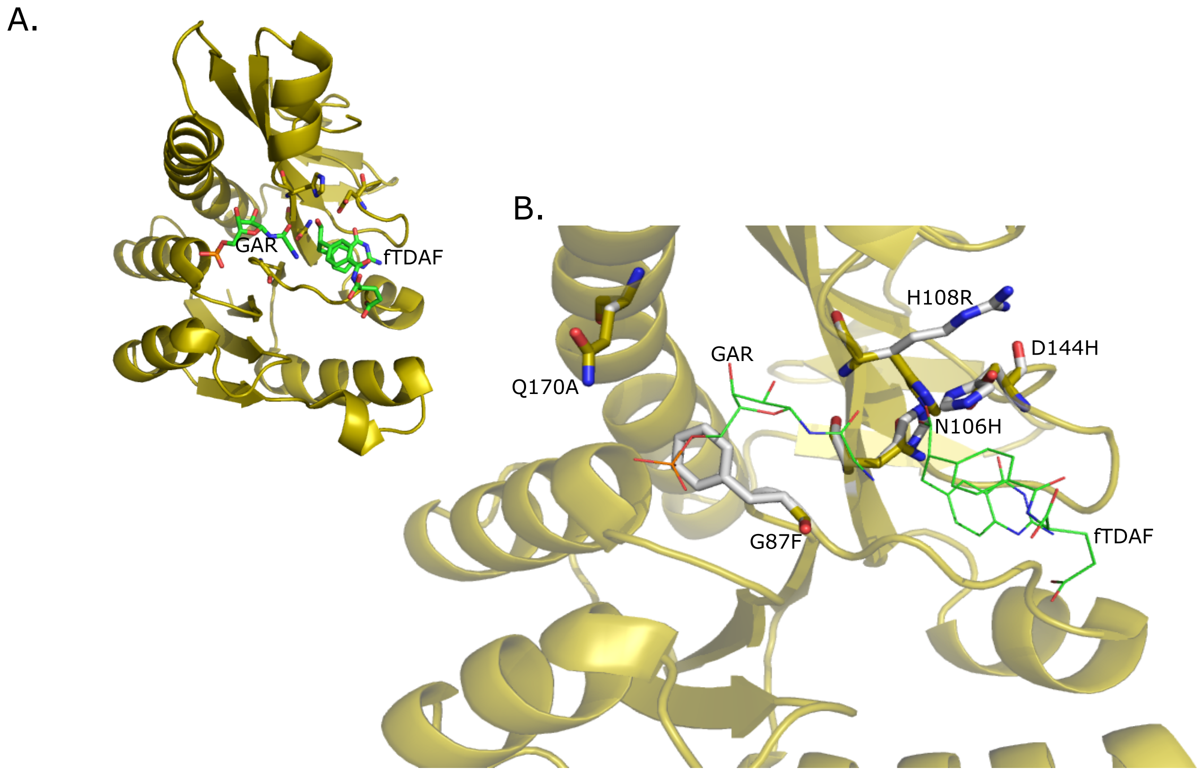


**Figure S1. Crystal structure of *E. coli* GART and its active site amino acids. A.** Strucutre of GART in complex with GAR and the fTHF analogue, 10-formyl-5,8,10-trideazafolic acid (fTDAF) (green sticks) (PDB 1C2T). Active site residues are shown in olive sticks. **B.** Substitutions of the active site residues. The modelled substitutions, G87A, G87F, N106H, H108R, D144H and Q170A are labelled and represented in silver sticks. The modelled mutations correspond to G147A, G147F, N168H, H170R, D229H and N253A mutations, respectively, in PvdF.

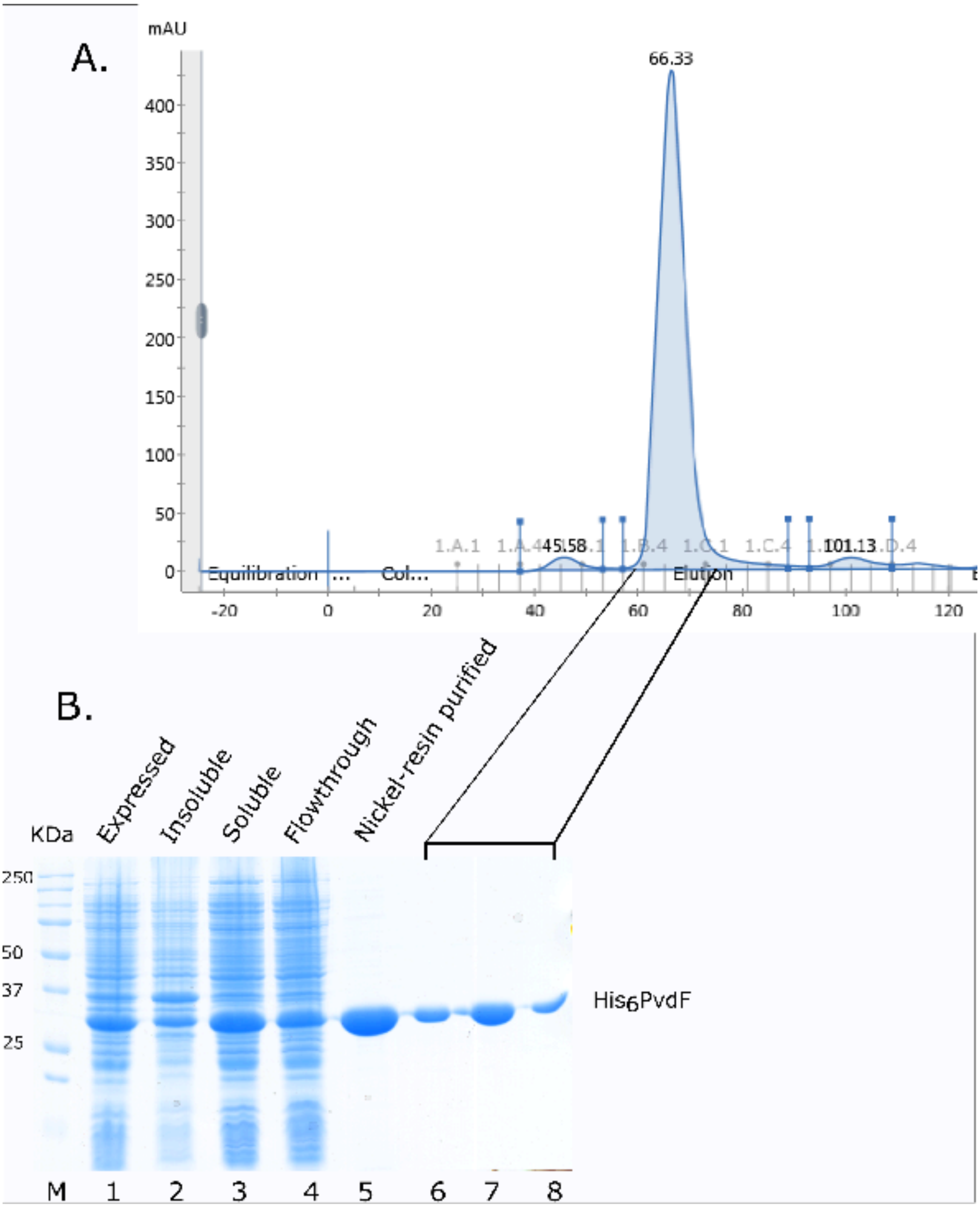


**Figure S2.** Purification of His$_6$PvdF using $Ni^{2+}$- affinity resin and size exclusion chromatography. *E. coli* BL21 (DE3) (pET-DUET*his$_6$pvdF*) was used to overexpress His$_6$PvdF. Following cell disruption, the soluble fraction was separated and used for purification. **A.** Chromatogram obtained during size exclusion chromatography (SEC) of semi-purified His$_6$PvdF. **B.** SDS gel of samples at different stages of purification. The Nickel-resin purified sample was used for SEC. The SEC fractions shown were pooled together and used for enzyme assays or snap frozen and stored at -80 °C for later use.

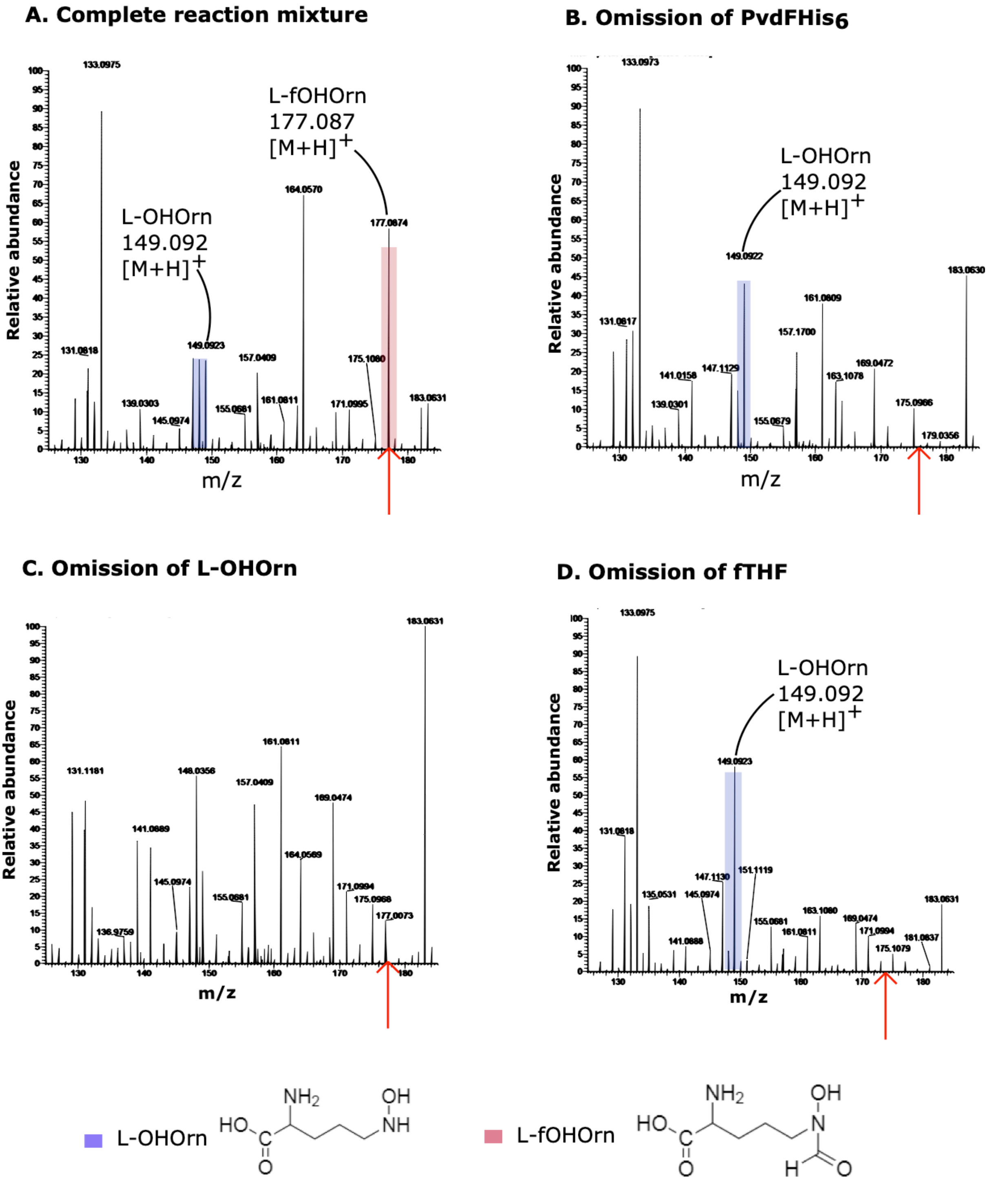


**Figure S3. PvdFHis$_6$ catalyses synthesis of fOHOrn from OHOrn and fTHF.** Reaction mixtures containing OHOrn, fTHF and PvdFHis$_6$ were incubated and analysed by direct injection mass spectrometry. Spectra present relative peak intensities of ionised molecules in a range of mass to charge ratios (m/z) from 125 to 185. **A.** PvdFHis$_6$ reaction showing peaks corresponding to OHOrn ([M+H]$^+$ 149.0921 +/- 3 ppm; blue) and fOHOrn ([M+H]$^+$ 177.0870 +/- 3 ppm; red). **B.** Negative control with omission of PvdFHis$_6$. **C.** Negative control with omission of OHOrn. Note the background peak at m/z 177.0566 is not related to the fOHOrn peak at m/z 177.0870 (delta m/z > 170 ppm). **D.** Negative control with omission of fTHF.

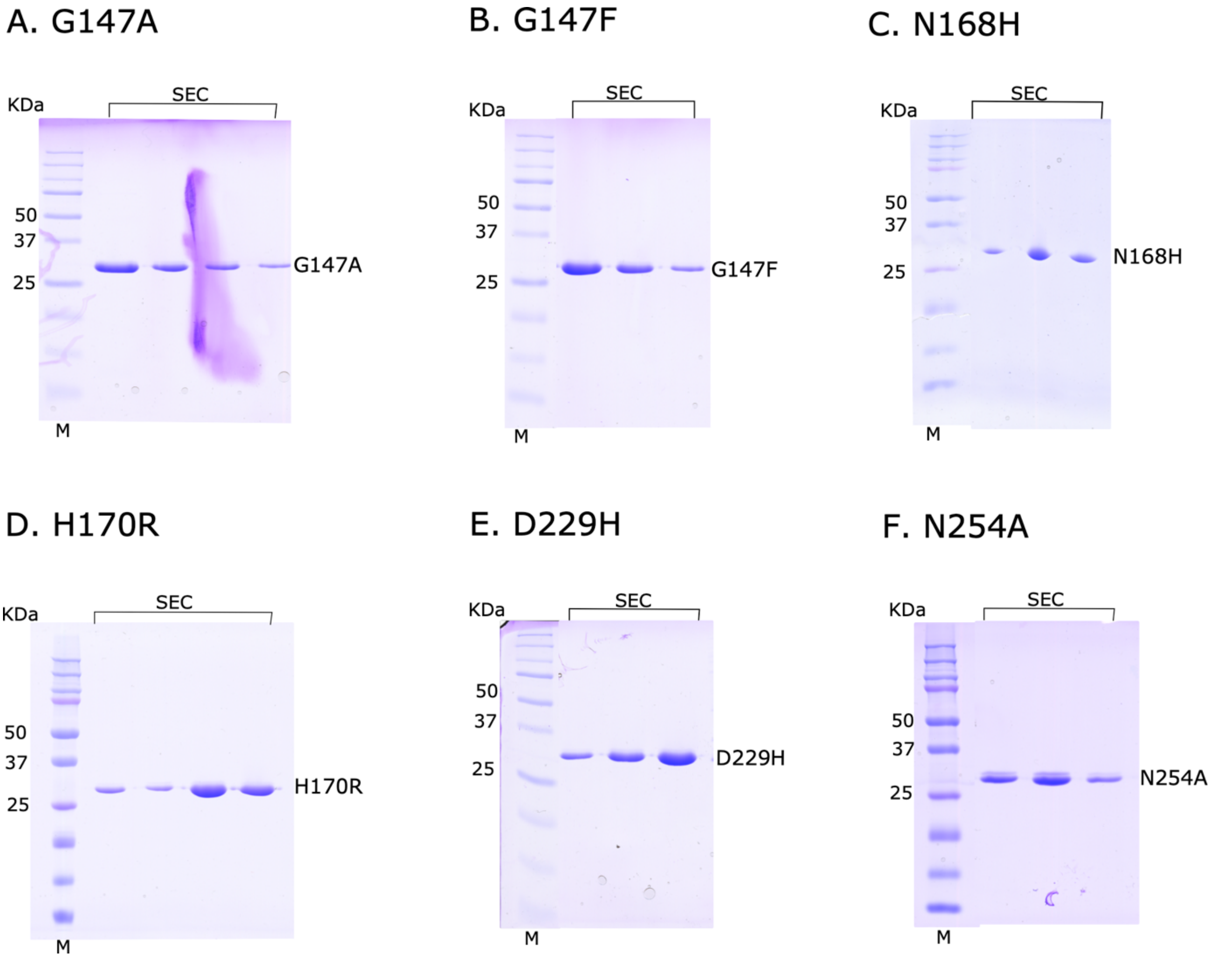


**Figure S4. Purification of PvdF mutant enzymes.** PvdF mutant variants were overexpressed in *E. coli* BL21(DE3) and cell disruption was carried out using sonication. The soluble fractions were loaded onto $Ni^{2+}$-resin to purify the proteins. The proteins were further purified using size exclusion chromatography. The eluted proteins were analysed using SDS-PAGE. The corresponding bands of each variant showed pure protein after SEC. The fractions for each variant were pooled together and used for enzyme assays or snap frozen and stored at -80 °C for later use. Full details of the purification protocol are provided in the manuscript.

A.

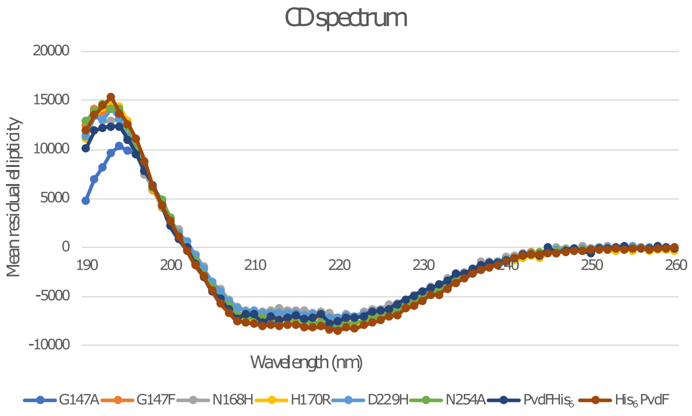


B.

| Protein | Alpha helix | Sheet | Coils |
|---|---|---|---|
| $His_6$PvdF | 0.31 | 0.14 | 0.54 |
| PvdF$His_6$ | 0.31 | 0.14 | 0.54 |
| G147A | 0.31 | 0.14 | 0.54 |
| G147F | 0.31 | 0.16 | 0.54 |
| N168H | 0.29 | 0.18 | 0.53 |
| H170R | 0.31 | 0.15 | 0.54 |
| D229H | 0.30 | 0.17 | 0.53 |
| N254A | 0.31 | 0.15 | 0.54 |

**Figure S5. Circular dichroism of PvdF and mutant variants.** Circular dichroism (CD) was used to analyse the secondary structure of wildtype and mutant PvdF. Three scans were carried out for each sample. The mean was calculated for each sample, the data were normalized with blank and the ellipticity was converted into mean residual ellipticity. **A.** CD spectra of PvdF wild type and mutant proteins plotted in mean residual ellipticity as a function of the far-UV region spectrum. **B.** The secondary structures were analysed using Dichroweb. The predicted secondary structure composition of the mutants was close to WTs showing no major changes in protein structure due to mutation.

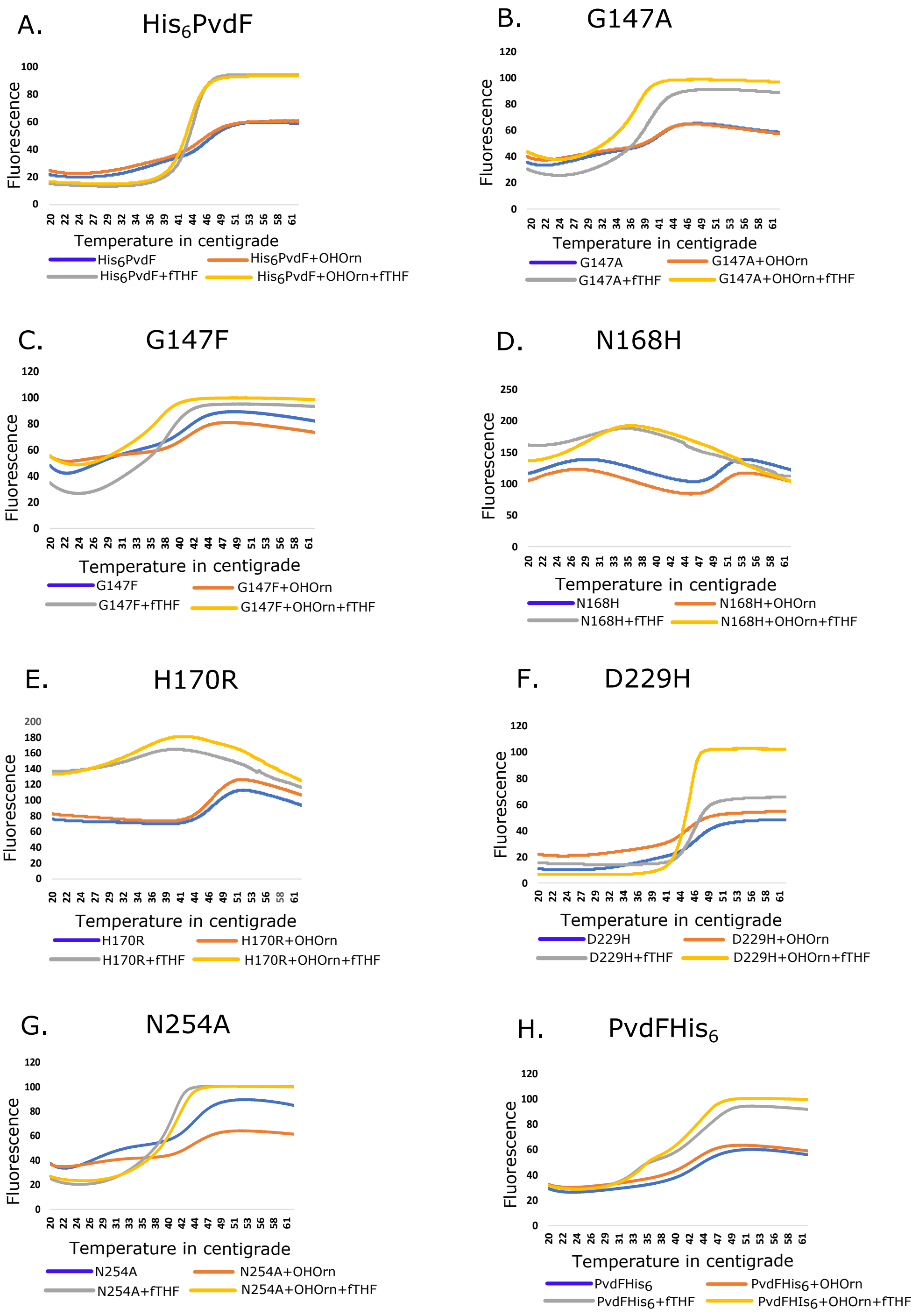


**Figure S6. Addition of OHOrn had no effect on protein melting.** The thermal stability tests of PvdF mutant enzymes and WT were carried out using SYPRO® orange in the presence and absence of substrates. The fluorescence was recorded at 580 nm using LightCycler® 480. The thermal stability curves are plotted in fluorescence as a function of temperature in centigrade. **A-G).** The protein melting curves of PvdF mutant enzymes and WT in the presence and absence of OHOrn and fTHF. The blue indicates protein with no substrate and the orange indicates protein with L-OHOrn. The yellow colour indicates protein in the presence of OHOrn and fTHF whereas the grey represents protein with fTHF. The blue and orange curves show similar pattern as the addition of OHOrn has no effect on protein stability whereas the yellow and grey are similar as the protein stability is increased with addition of fTHF with a higher shift in fluorescence amplitude.

## References

McMorran BJ, Kumara HMCS, Sullivan K & Lamont IL (2001) Involvement of a transformylase enzyme in siderophore synthesis in *Pseudomonas aeruginosa*. *Microbiology* **147**: 1517-1524.

Schweizer HP (1991) Improved broad-host-range *lac*-based plasmid vectors for the isolation and characterization of protein fusions in *Pseudomonas aeruginosa*. *Gene* **103**: 97-92.

Studier FW & Moffatt BA (1986) Use of bacteriophage T7 RNA polymerase to direct selective high-level expression of cloned genes. *J Mol Biol* **189**: 113-130.
